# Binaural speech intelligibility for combinations of noise, reverberation, and hearing-aid signal processing

**DOI:** 10.1371/journal.pone.0317266

**Published:** 2025-01-15

**Authors:** James M. Kates, Mathieu Lavandier, Ramesh Kumar Muralimanohar, Emily M. H. Lundberg, Kathryn H. Arehart

**Affiliations:** 1 Deptartment of Speech, Language, and Hearing Sciences, University of Colorado, Boulder, Colorado, United States of America; 2 ENPTE, Ecole Centrale de Lyon, CNRS, LTDS, UMR5513, Vaulx-en-Velin, France; 3 Department of Communication Sciences and Disorders, University of Northern Colorado, Greeley, Colorado, United States of America; University of Southern Mississippi, UNITED STATES OF AMERICA

## Abstract

Binaural speech intelligibility in rooms is a complex process that is affected by many factors including room acoustics, hearing loss, and hearing aid (HA) signal processing. Intelligibility is evaluated in this paper for a simulated room combined with a simulated hearing aid. The test conditions comprise three spatial configurations of the speech and noise sources, simulated anechoic and concert hall acoustics, three amounts of multitalker babble interference, the hearing status of the listeners, and three degrees of simulated HA processing provided to compensate for the noise and/or hearing loss. The impact of these factors and their interactions is considered for normal-hearing (NH) and hearing-impaired (HI) listeners for sentence stimuli. Both listener groups showed a significant reduction in intelligibility as the signal-to-noise ratio (SNR) decreased, and showed a reduction in intelligibility in reverberation when compared to anechoic listening. There was no significant improvement in intelligibility for the NH group for the noise suppression algorithm used here, and no significant improvement in intelligibility for the HI group for more advanced HA processing algorithms as opposed to linear amplification in either of the two acoustic spaces or at any of the three SNRs.

## Introduction

Binaural speech intelligibility is affected by many factors including the spatial configuration of the speech and noise sources, the room acoustics, the type and amount of interference, the hearing status of the listeners, and the processing provided to compensate for the noise and/or hearing loss. Intelligibility also depends on the interactions of these factors that occur in everyday listening situations. This paper presents measurements of the impact of these factors both alone and in combination using simulated acoustic environments and hearing-aid (HA) processing. The main objective of this study was to measure binaural speech intelligibility over a wide range of realistic processing conditions and interactions, and an additional objective was to validate our findings against studies that considered fewer conditions or limited interactions.

The benefit of spatial separation of the speech and interference, or spatial release from masking, is strongest under anechoic conditions. When the azimuth of an interfering source differs from that of the speech, it provides useful cues that improve speech intelligibility [1–3]. One cue is the head shadow, in which the ear closest to the speech target exhibits an increase in the speech sound pressure at high frequencies while the opposite ear experiences a decrease [4]. The azimuths of the target and interference also introduce interaural time delays (ITD) which, according to the equalization cancellation (EC) theory, allow partial auditory cancellation of the interference [5, 6]. These cues can also be used by HI listeners, although the binaural benefit tends to be reduced with increasing hearing loss [7–9]. A related effect is better ear glimpsing, where intelligibility in fluctuating interference is dominated by the ear having the better instantaneous signal-to-noise ratio (SNR). Glimpsing requires that the speech in the noise gaps be audible, so this effect also tends to be reduced with increased hearing loss [10] unless sufficient amplification is provided as compensation [11].

Several studies have found a decrease in intelligibility with increasing reverberation time (RT). For example, a significant reduction in intelligibility between anechoic and reverberant speech was found for both NH and HI listeners [12], but increasing the RT from 0.5 to 1.0 s did not lead to any additional reduction in intelligibility. However, other studies [8, 13, 14] have found significant correlations between increased RT and decreased speech intelligibility for both sentence and isolated word test materials. Another aspect of reverberation is the direct-to-reverberant ratio (DRR), which decreases the further the target speech source is moved from the listener within a room. For experiments that keep the room dimensions and the source and listener positions constant but vary the wall sound absorption, the RT and DRR will be highly correlated since increasing the RT decreases the DRR [15].

Combining noise with reverberation further reduces speech intelligibility. At the phoneme level, intelligibility decreases with increasing noise and reverberation for NH and for listeners having both mild and moderately-severe hearing losses [8]. The same study [8] also found an interaction between noise and reverberation in that the detrimental reverberation effects were amplified by the addition of noise. Other studies have observed similar results [15–17].

Finally, it is important to consider the interaction of HA processing with noise and room acoustics. Wide dynamic-range compression (WDRC) is characterized by the speed with which the system reduces gain in response to increases in the signal intensity (attack time) and increases gain in response to reductions in the signal intensity (release time). In a study involving HI listeners [13], it was found that intelligibility decreased with increased RT and decreased with shorter WDRC release times. They also observed a significant interaction between WRDC and RT; faster release times caused a greater reduction in intelligibility at the longer RTs. A related study [18] found similar effects of decreased intelligibility with increased reverberation time and decreased SNR. They also found an interaction of WDRC with spectral subtraction; the noise suppression was beneficial when combined with fast WDRC but detrimental when combined with slow WDRC. The presence of the hearing aid itself may also reduce intelligibility for speech in noise and reverberation [19]. In that study NH subjects were presented with speech in noise with a small amount of reverberation while listening through behind-the-ear (BTE) HAs programmed to have a flat frequency response. The result was that even without WDRC the HAs reduced spatial release from masking compared to the unaided condition.

Speech intelligibility for HI listeners under realistic listening conditions has also been investigated for speech combined with noise and WDRC. For a virtual listening system with a simulated HA having an attack time of 5 ms and a release time of 100 ms [20], intelligibility was found to be higher for real-world modulated background noises than for stationary noise, and was higher for the aided condition than for the unaided condition. Another study [21] looked at intelligibility for HI listeners using WDRC HAs processing speech in babble at SNRs ranging from 0 dB up to quiet in a small room. They found that for omnidirectional HA microphones, fast WDRC (attack time = 12 ms, release time = 70 ms) gave lower intelligibility than slow WDRC (attack time = 30 ms, release time = 4000 ms) at low SNRs but similar results in quiet.

More complicated HA processing has also been investigated, one example being WDRC combined with frequency compression. In frequency compression, higher frequencies are shifted lower to regions of better audibility in the impaired ear. Measurements of binaural cues were obtained [22] for a commercial HA programmed with syllabic WDRC (attack time = 1 ms, release time = 50 ms) and frequency compression for a severe high-frequency hearing loss. The study found that WDRC caused distortions of interaural level differences (ILD) and that frequency compression caused distortions in high-frequency interaural timing differences (ITD) and reduced interaural coherence. The authors hypothesize that the altered relationships between ILDs and ITDs impact binaural perception, but subject data are not provided.

The main objective in this paper is to explore speech intelligibility for a variety of factors and interactions using realistic room and HA simulations. The paper focuses on the spatial configuration of the speech and noise sources, room acoustics, SNR for multi-talker babble, hearing status, and HA processing. The HA processing is represented by a combination of algorithms that includes linear amplification, WDRC, noise suppression based on spectral subtraction, and frequency compression using a sinusoidal modeling approach. The linear amplification condition allows the comparison of results between the NH and HI listener groups for differences in spatial configuration, reverberation, and SNR, while the remaining conditions and interactions are analyzed separately for the two listener groups. The experimental design is described in the Methods section below, followed by the results and statistical analysis, discussion of the results, and the conclusions.

## Methods

**Fig 1:**
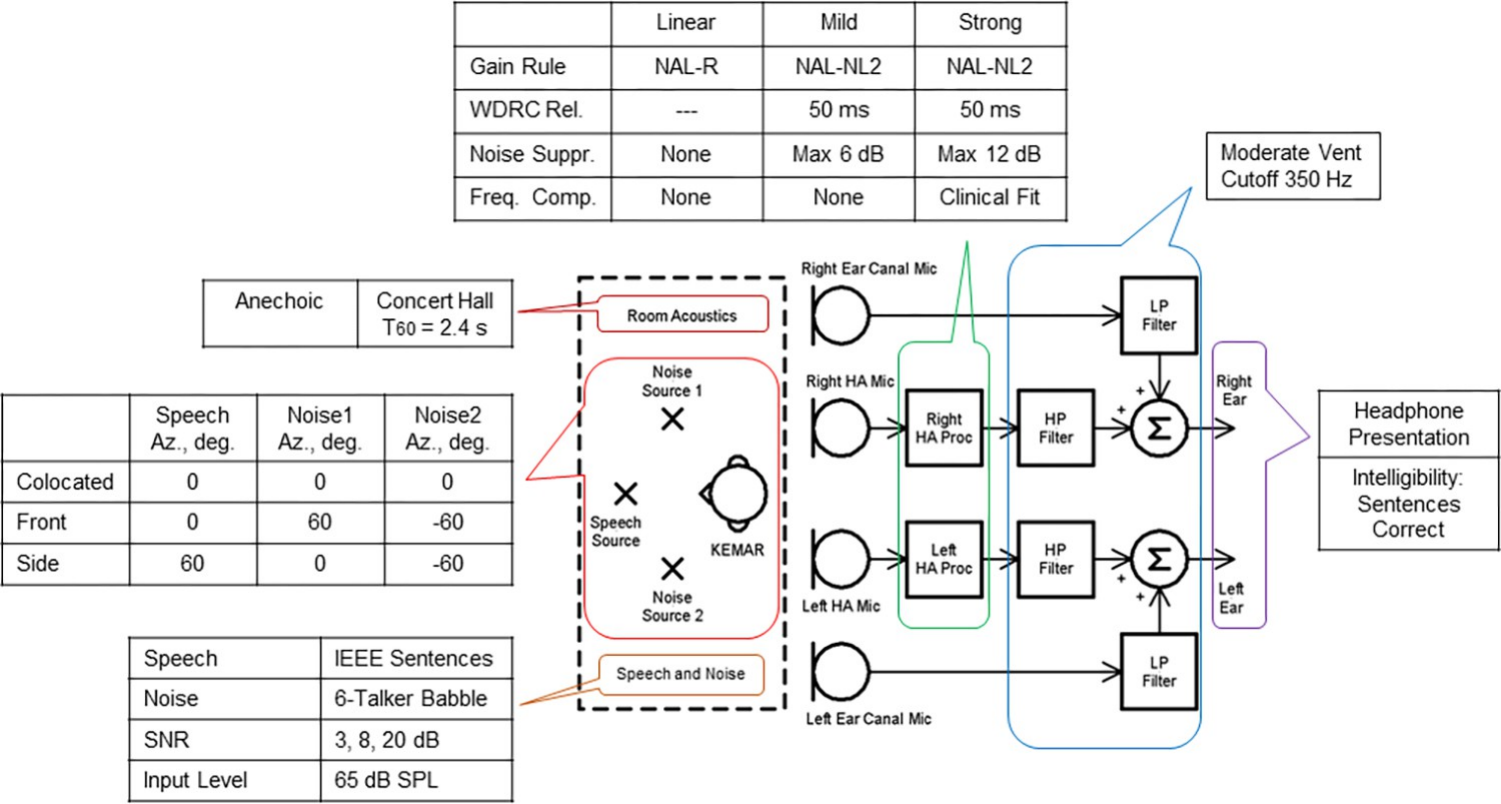
Overview of the experimental design. The experimental design uses binaural headphone stimulus presentation. The experiment combines a simulated acoustic space, dummy-head head-related impulse response (HRIR) measurements, and bilateral hearing-aid (HA) simulations. The processing parameters used in generating the stimuli are indicated in the embedded tables. The processing for the HI listeners used the tabulated sets of linear, mild, and strong parameter values; the NH listeners were presented only with the three noise suppression settings.

The goal of the experiment was to acquire speech intelligibility scores for a variety of listening situations. An overview of the experimental design is shown in Fig 1; the experiment used dummy-head head-related impulse response (HRIR) recordings and binaural headphone stimulus presentation. Two simulated acoustic spaces, three speech and noise spatial configurations, three SNRs, and three simulated HA processing settings were used, giving a total of 54 listening conditions. Both listeners with normal hearing and listeners with hearing loss were recruited to participate in the study. Recruitment of participants occurred between November 22, 2021, and April 26, 2023.

### Room simulation

Two room simulations, an anechoic space (denoted as anechoic) and a concert hall (denoted as hall), were created using the University of Minnesota Multi-Sensory Perception (MSP) Laboratory facility, which is part of the Center for Applied and Translated Sensory Science (CATSS). The simulations used a loudspeaker array located in a semi-anechoic chamber which also contained video projectors, cameras, and the electronics associated with signal presentation and recording. The signals to each loudspeaker were controlled by a virtual-image room simulation system that includes the propagation delays and power-law attenuation for each virtual image. The loudspeaker array provides a 2-dimensional virtual image room simulation having loudspeakers located at 10-deg increments in the azimuthal plane surrounding the dummy head position, with one of the azimuthal loudspeakers removed to allow door access. The azimuth of 0 deg was defined as being directly in front of the dummy head used for the recordings.

Each virtual image was assigned to the closest loudspeaker in the array. The default anechoic response of the free-field array was used to provide KEMAR manikin [23] HRIR recordings at the 10-deg azimuth increments; reflections from the extraneous surfaces in the chamber were at least 25 dB below the peaks of the recorded anechoic HRIR measurements. A concert hall having RT = 2.4 s (DRR = 4.3 dB for colocated speech and noise sources in front and signals recorded at the HA microphones) was also simulated using virtual images. The source-to-receiver distances for both the anechoic and concert hall conditions were 1.9 m, which was the closest allowed by the loudspeaker configuration. The room simulation does not include atmospheric sound absorption; this absorption was added to the recorded room impulse responses [24].

The anechoic or simulated room responses used to create the HA input signals were recorded using commercial HA microphones mounted in the front and rear positions of BTE shells placed above the left and right ears of KEMAR. The left and right front microphones were used to generate the HA input signals for the listener experiment. Ear-canal responses were also recorded simultaneously from both ears of KEMAR using G.R.A.S. 40AG microphones (G.R.A.S. Sound and Vibration, Holte, Denmark). The recorded microphone signals thus included the simulated room reverberation (if present) and the KEMAR HRIR associated with each loudspeaker location.

### Hearing aid simulation

The bilateral HA simulation shown in Fig 1 combined the recorded KEMAR microphone responses with off-line HA processing; the HA processing provided independent operation at the two ears [13, 25, 26]. The inputs to the left and right hearing aids were the speech and noise stimuli convolved with the reverberation and the KEMAR HA microphone and ear canal responses described in the section above. Frequency analysis was implemented using a six-channel linear-phase finite impulse response (FIR) filterbank having band center frequencies at (250, 500, 1000, 2000, 4000, and 6000) Hz. The HA processing within each frequency band used a series configuration, with noise suppression first followed by WDRC and then frequency compression.

For both NH and HI listeners, noise suppression was implemented using an adaptive Wiener filter with the noise estimate based on the root mean squared (RMS) average signal level computed over the duration of the stimulus [27]. For the HI listeners, compensation for loss of speech audibility was provided in two ways for each listener depending on the condition: 1) linear amplification using the NAL-R gain rule [28] or 2) wide dynamic-range compression (WDRC) using the NAL-NL2 procedure [29] with gains computed separately within each frequency band. High frequencies for the HI listeners were shifted lower in frequency using a frequency compression algorithm based on sinusoidal modeling [30]; frequencies below the cutoff frequency were passed through the system without modification while higher frequencies were shifted lower to fit into the impaired auditory bandwidth. The HA receiver response was bypassed to provide the widest possible output signal bandwidth, and a broadband time delay of 10 ms was added to the HA output to approximate the delays found in commercial devices.

Three processing conditions, linear, mild, and strong, were provided in the HA simulation used for the HI listeners. The processing parameters for HI listeners were adjusted according to the table at the top of Fig 1. The NAL-R amplification, NAL-NL2 compression, and frequency lowering were fit to the individual audiograms [31]. The same Wiener filter spectral subtraction noise suppression processing was used for both the HI and NH listeners. The NH subject group received 0-dB flat amplification with different degrees of noise suppression but without WDRC or frequency lowering; thus linear processing for the NH group indicates no noise suppression, mild indicates noise suppression having a maximum of 6 dB signal attenuation, and strong indicates noise suppression having a maximum of 12 dB signal attenuation.

The final step in the HA simulation was the earmold vent. The vent acts as an acoustic filter that affects the hearing-aid output and ear-canal signals [32, 33]. A complementary pair of 2-pole infinite impulse response (IIR) highpass and lowpass Butterworth filters was implemented in the simulation, with the highpass filter applied to the HA output and the lowpass filter applied to the signal recorded in the manikin ear canal. The cutoff frequency of the filters was 350 Hz, which represents a moderate vent having a radius of 0.6 cm [34].

### Listeners

The participants in the experiment comprised a group of 15 younger adults (mean age 22.23 years, range 19 to 28 years) with normal hearing and a group of 15 older adults (mean age 77.81 years, range 57 to 84 years) with bilateral mild to moderately-severe sensorineural hearing loss. Normal hearing was defined as air conduction thresholds being 25 dB HL or less measured bilaterally across test frequencies [35]. Thresholds for the HI listeners were symmetrical, defined as a threshold difference across ears of less than 20 dB if the difference occurred at just one test frequency or less than 15 dB for differences occurring at two or more test frequencies; this criterion is consistent with criteria reported in the literature [36].

**Fig 2:**
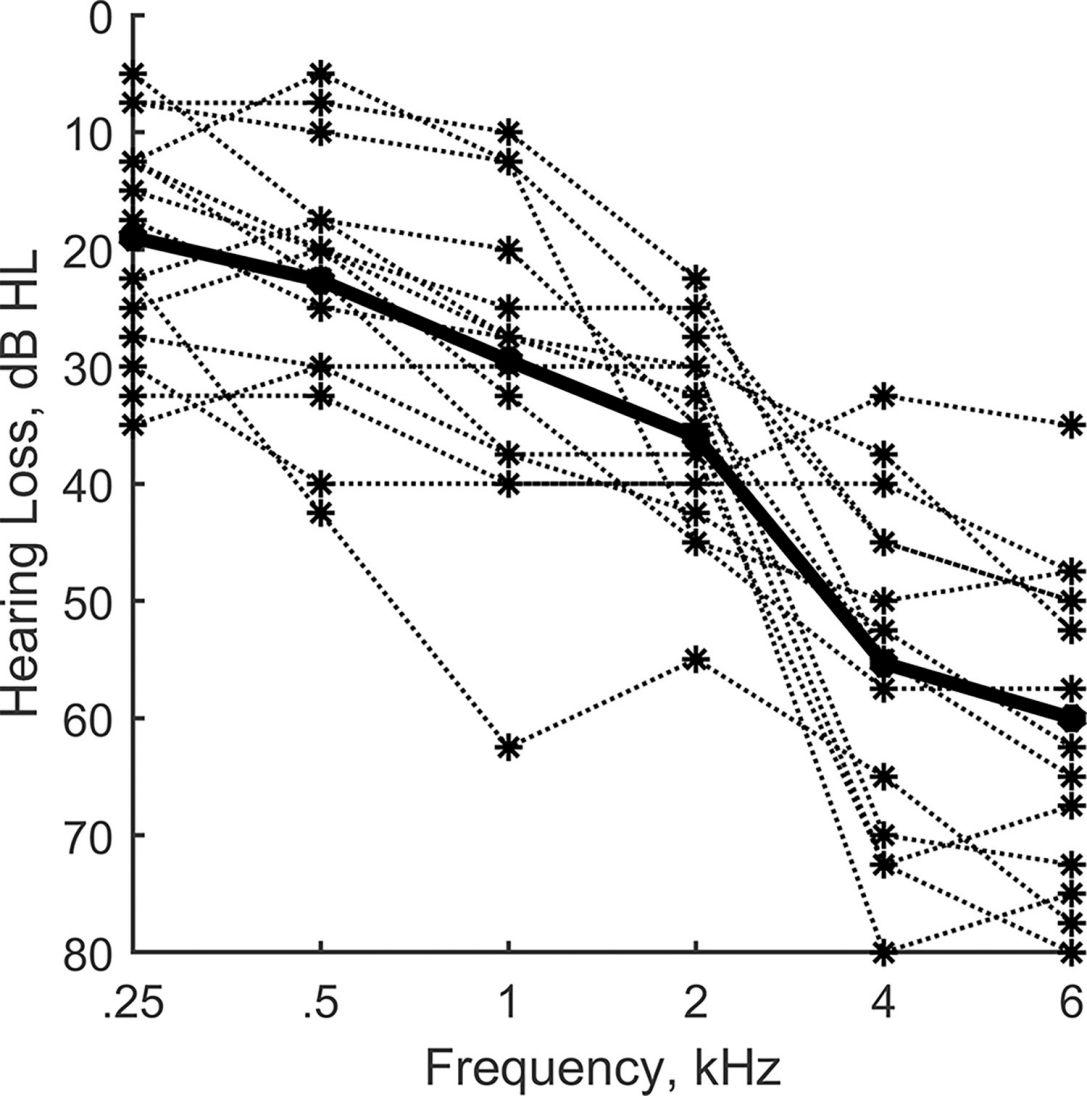
Audiograms for the HI listeners. Individual audiograms averaged across the two ears are indicated by the dotted lines, and the average audiogram for the HI group is indicated by the heavy solid line.

The audiograms for the individual HI listeners averaged across left and right ears are plotted in Fig 2 using dotted lines, and the group average is indicated by the solid black line. Tympanograms for all subjects showed normal peak pressure and static admittance bilaterally [37]. The average age of the listeners in the NH group was 22.3 years (range 19 to 28) and in the HI group was 62.5 years (range 57 to 84). All listeners were native speakers of English and all passed the Montreal Cognitive Assessment [38] with a score of at least 26. The experimental protocol was approved by the University of Colorado Institutional Review Board and all testing took place at the University of Colorado Boulder. Written informed consent was obtained for all participants. Listeners were reimbursed for their participation.

### Stimuli

The speech stimuli were low-context sentences from the Institute of Electrical and Electronics Engineers (IEEE) corpus [39]. The sentences were spoken by fifteen male and eighteen female talkers [40], with the combination of talker, list, and sentence within the list chosen at random for each processed sentence presented to each listener. A subset of 620 sentences was used to avoid materials having speech production issues [41]. The speech was mixed with two noise sources at SNRs of 3, 8, and 20 dB [42, 43]. Each noise source was a segment of six-talker babble from the Connected Speech Test (CST) [44]. The babble comprising each of the two noise sources was selected at random without replacement from one of nine segments and combined with each randomly selected sentence. The SNR was calculated as the ratio of the power of the speech signal averaged across the left and right HA microphones to the power of the combined noise signals averaged across the two microphones.

In continuous discourse, the reverberant tail from a preceding sentence will partially mask the onset of the target sentence. This effect was reproduced in the test stimuli by preceding the test sentence with a time-reversed version of the same sentence, with the time-reversed and test sentences separated by a 200-ms gap. The duration of the interfering noise was that of the combined time-reversed plus target speech. After reverberation was applied to both the speech and the noise, the preceding sentence with its associated noise was pruned, leaving the noisy target sentence with its onset masked by the reverberant tail from the time-reversed noisy sentence.

Three spatial configurations were used in the experiment, as listed in Fig 1. These configurations were chosen for consistency with previous studies [2, 45, 46]. A positive azimuth moved the sound source to the right when looking down on KEMAR. The front configuration was simulated as speech coming from the 0-deg loudspeaker position (directly in front of KEMAR) combined with two different babble segments symmetrically simulated as coming from the +60 deg and -60 deg loudspeaker positions. The simulated colocated configuration moved both babble segments to the 0-deg loudspeaker position, so the reproduced noise was the sum of two separate segments. The simulated side configuration moved the speech to the +60 deg loudspeaker position and placed the babble segments at the 0 and -60 deg loudspeaker positions, so the speech came from the right with one noise source directly in front and the second noise source located to the left. For the front and colocated configurations the SNR was approximately the same at the two ears, while for the side configuration the SNR at the right ear was approximately 8.7 dB higher than the SNR at the left ear.

The experiment comprised two simulated rooms × three SNRs × 3 spatial configurations × 3 HA processing settings, giving a total of 54 conditions. Each condition was presented 10 times, each time with different randomly-selected talker, sentence, and babble segments, giving a total of 540 sentences scored for each listener. Each listener heard a unique random ordering of the IEEE sentence, talker, babble segments, room, spatial configuration, and HA processing. The participants responded verbally. The tester recorded the responses by indicating which words were repeated correctly via a graphical user interface (GUI) with buttons for each word.

### Stimulus presentation

Listeners were seated in a sound-isolation booth. Participants heard the processed speech presented through Sennheiser HD-25 headphones via a Tucker Davis Technologies (TDT) RX8 processing system which included a TDT PA5 attenuator and a TDT HB7 headphone buffer. The stimulus selection and playout system were controlled using custom MATLAB scripts. Participants completed the experiment over two visits of 1.5–2 hours each. The visits included the audiological and cognitive tests, consent forms, a short training session, and the speech intelligibility testing. The training portion comprised ten sentences that represented a subset of the conditions tested, and the scored intelligibility testing was completed after the training. The IEEE sentences were scored in terms of keywords correct which were then converted to sentences correct for the analysis in this paper.

## Results

The factors in the experimental design comprised the room (anechoic and concert hall), spatial configuration (Config: colocated, front, and side), SNR (3, 8, and 20 dB), hearing loss (HL: NH and HI), and HA processing (Process: linear, mild, and strong) along with the interactions of these factors. These factors are summarized in Fig 1. Note that the processing options for NH differ from those used for HI. Linear is similar for both subject groups in that there is no nonlinear processing and audibility is maintained via NAL-R amplification matched to the audiogram. However, the mild and strong processing for the NH group comprised up to 6 dB or 12 dB of noise suppression, respectively, while the mild processing for the HI group had WDRC and up to 6 dB of noise suppression and the strong processing for the HI group had WDRC, up to 12 dB of noise suppression, and frequency lowering. The amount of nonlinear distortion inherent in the HI mild and strong conditions is thus greater than for the mild and strong NH conditions.

### Experimental conditions and sentence intelligibility

Linear Mixed Effects Regression (LMER) models [47] were created to analyze the effects of the factors and their interactions on sentence-level speech intelligibility. The models were implemented in R v4.3.1 [48] using the lmer() function [49] with participant as a random intercept. All data were averaged over the 10 repetitions of each of the 54 conditions for each subject prior to performing the analysis.

Since the NH and HI groups received different combinations of nonlinear processing, the linear processing condition is the only one that allows for direct comparisons between the two groups. The results of the Type III tests from the LMER analysis are presented in Table 1 for linear processing. The main effects of room, SNR, spatial configuration, and HL group are all significant at the *p* < 0.001 level, as is the interaction of SNR and spatial configuration. The interactions of SNR with HL group and room × SNR × HL group are significant at the *p* < 0.01 level, and the interaction of room with HL group is significant at the *p* < 0.05 level.

**Table 1:** LMER results for sentence correct scores, linear processing.

	Df	*F*	*p*
Room	1	194.8493	< 0.0001***
SNR	2	511.2071	< 0.0001***
Spatial Configuration	2	78.9997	< 0.0001***
HL	1	40.1749	<0.0001***
Room x SNR	2	2.9255	0.0546^
Room x Spatial Configuration	2	4.6602	0.0099**
Room x HL	1	4.2624	0.0395*
SNR x Spatial Configuration	4	16.8800	<0.0001***
SNR x HL	2	6.9856	0.0010**
Spatial Configuration x HL	2	2.8751	0.0574^
Room x SNR x Spatial Configuration	4	1.6251	0.1667
Room x Spatial Configuration x HL	2	0.7330	0.4810
SNR x Spatial Configuration x HL	4	1.5150	0.1966
Room x SNR x HL	2	6.9343	0.0011**
Room x SNR x Spatial Configuration x HL	4	0.3072	0.8732

The analysis is for the main effects and interactions of spatial configuration, room, SNR, and hearing-loss group (HL) for linear processing.

*** indicates significance at the 0.0001 level

** at the 0.001 level

* at the 0.05 level, and

^ close to 0.05 level.

Because HL group was highly significant and there were also several significant interactions with HL group, separate analyses were conducted for the NH and HI groups. The results for the NH group are presented in Table 2. The factors of room, SNR, spatial configuration, and noise suppression are all significant at *p* < 0.001, as is the interaction of SNR × spatial configuration. The interaction of SNR × noise suppression is significant for the NH group at *p* < 0.05. The results for the HI group are presented in Table 3. The factors of room, SNR, and spatial configuration are significant at *p* < 0.001, but HA processing is not significant (*p* > 0.05). The interactions of room × SNR and SNR × spatial configuration are both significant at the *p* < 0.001 level. Also, the interaction of room × spatial configuration is significant at *p* < 0.01 for the HI group.

**Table 2:** LMER results for sentence correct scores, NH listeners.

	Df	*F*	*p*
Room	1	224.4828	< 0.0001***
SNR	2	974.1080	< 0.0001***
Spatial Configuration	2	160.5009	< 0.0001***
Noise Suppression	2	7.5543	0.0006**
Room x SNR	2	0.8042	0.4478
Room x Spatial Configuration	2	2.8387	0.0591^
Room x Noise Suppression	2	2.9936	0.0507^
SNR x Noise Suppression	4	2.6570	0.0319*
SNR x Spatial Configuration	4	50.0227	< 0.0001***
Spatial Configuration x Noise Suppression	4	0.3611	0.8363
Room x SNR x Spatial Configuration	4	2.0425	0.0867
Room x SNR x Noise Suppression	4	0.9921	0.4110
Room x Spatial Config x Noise Suppression	4	0.4706	0.7574
SNR x Spatial Config x Noise Suppression	8	0.8982	0.5174
Room x SNR x Spatial Config x Noise Suppress.	8	0.7928	0.6091

The analysis is for the main effects and interactions of spatial configuration, room, SNR, and noise reduction in the NH Group.

*** indicates significance at the 0.0001 level

** at the 0.001 level

* at the 0.05 level, and

^ close to 0.05 level.

**Table 3:** LMER results for sentence correct scores, HI listeners.

	Df	*F*	*p*
Room	1	303.8599	< 0.0001***
SNR	2	640.7432	< 0.0001***
Spatial Configuration	2	95.5976	< 0.0001***
Processing	2	2.0206	0.1333
Room x SNR	2	25.8194	< 0.0001***
Room x Spatial Configuration	2	4.983	0.0071*
Room x Processing	2	0.8221	0.4400
SNR x Processing	4	0.142	0.9665
SNR x Spatial Configuration	4	19.7701	< 0.0001***
Spatial Configuration x Processing	4	0.5669	0.6867
Room x SNR x Spatial Configuration	4	1.7094	0.1460
Room x SNR x Processing	4	0.4051	0.8050
Room x Spatial Config x Processing	4	0.4905	0.7428
SNR x Spatial Config x Processing	8	1.7636	0.0809
Room x SNR x Spatial Config x Processing	8	0.783	0.6178

The analysis is for the main effects and interactions of spatial configuration, room, SNR, and HA processing in the HI Group.

*** indicates significance at the 0.0001 level

** at the 0.001 level

* at the 0.05 level, and

^ close to 0.05 level.

**Fig 3:**
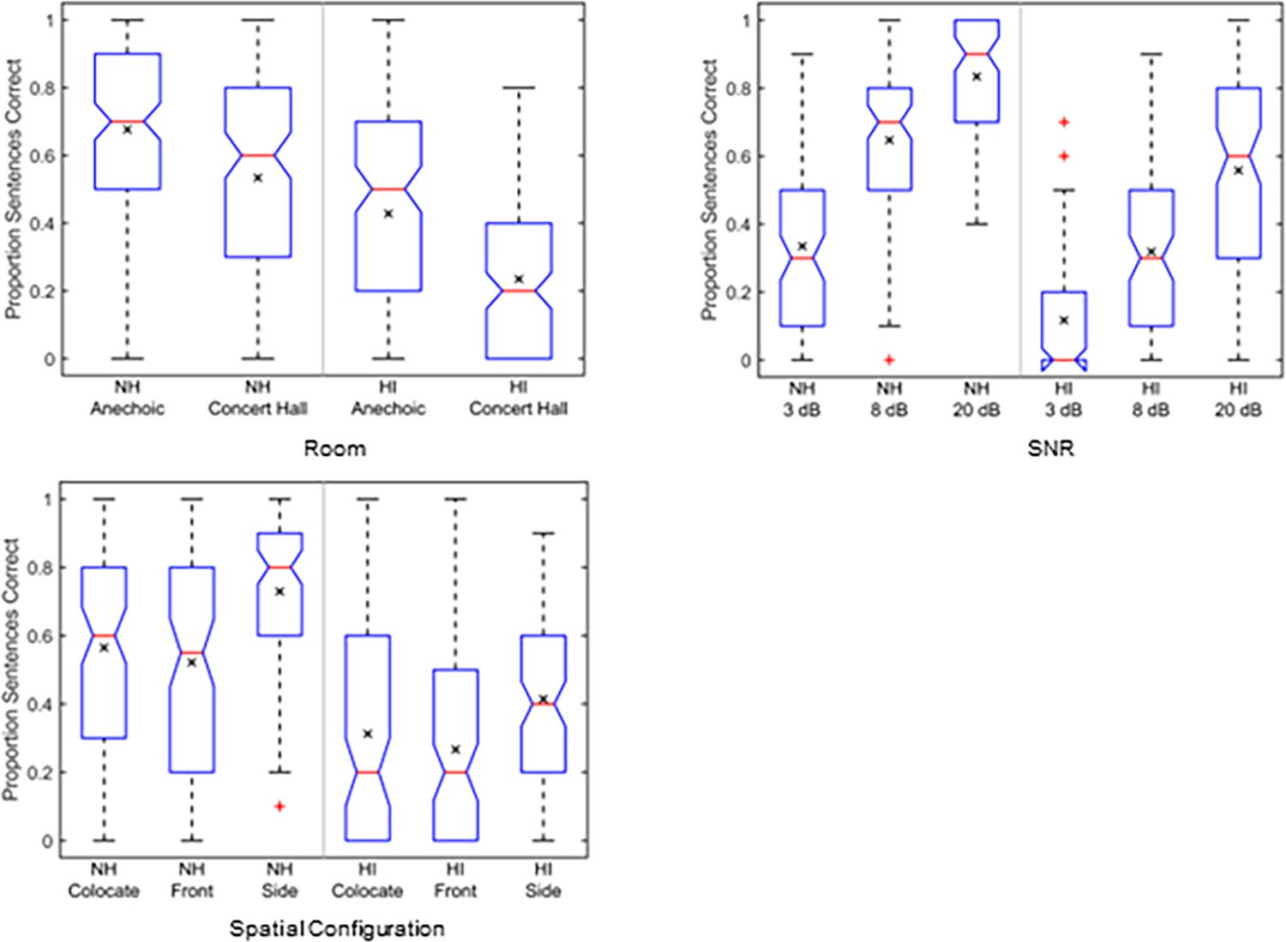
Box plots for the factors of room, spatial configuration, and SNR. The box plots are computed separately for the NH and HI listener groups for the linear processing condition. The means are given by the × within each box. The processing for the NH listeners comprised flat 0 dB amplification, while that for the HI listeners comprised NAL-R linear amplification computed for the individual audiograms. Data are averaged over repetitions for each subject.

The box plots in Fig 3 show distributions of intelligibility scores for linear amplification measured as proportion sentences correct. The factors are room, SNR, and spatial configuration for the two groups of listeners. Intelligibility for the simulated room is shown in the top left panel. Pair-wise comparisons computed using Bonferroni adjustments [50] show that intelligibility is lower for the concert hall than for the anechoic condition for both groups of subjects (*p* < 0.001, 1.02 ≤ *d* ≤ 1.38), and intelligibility is lower for the HI group than for the NH group in both environments (*p* < 0.001, 1.38 ≤ *d* ≤ 1.78), where *d* is the effect size (Cohen’s *d*). Intelligibility as a function of SNR is shown in the top right panel. Intelligibility is lower for the SNR of 3 dB compared to 8 dB, and is lower at 8 dB compared to 20 dB, for both groups of subjects (*p* < 0.001, 1.34 ≤ *d* ≤ 2.24). Intelligibility for the HI group is lower than for the NH group at all three SNRs (*p* < 0.001, 1.56 ≤ *d* ≤ 2.35). Intelligibility as a function of spatial configuration is shown in the lower left panel. Intelligibility for the side configuration is higher than for the colocated or front configurations for both listener groups (*p* < 0.001, 0.73 ≤ *d* < 1.49). There is no significant difference in intelligibility between the front and colocated conditions for either the NH group (*p* = 0.569, *d* = 0.31) or the HI group (*p* = 0.437, *d* = 0.33). Intelligibility for the HI group is lower than for the NH group for all three spatial configurations (*p* < 0.001, 1.81 ≤ *d* ≤ 2.25).

### Pairwise comparisons: NH Group

**Fig 4:**
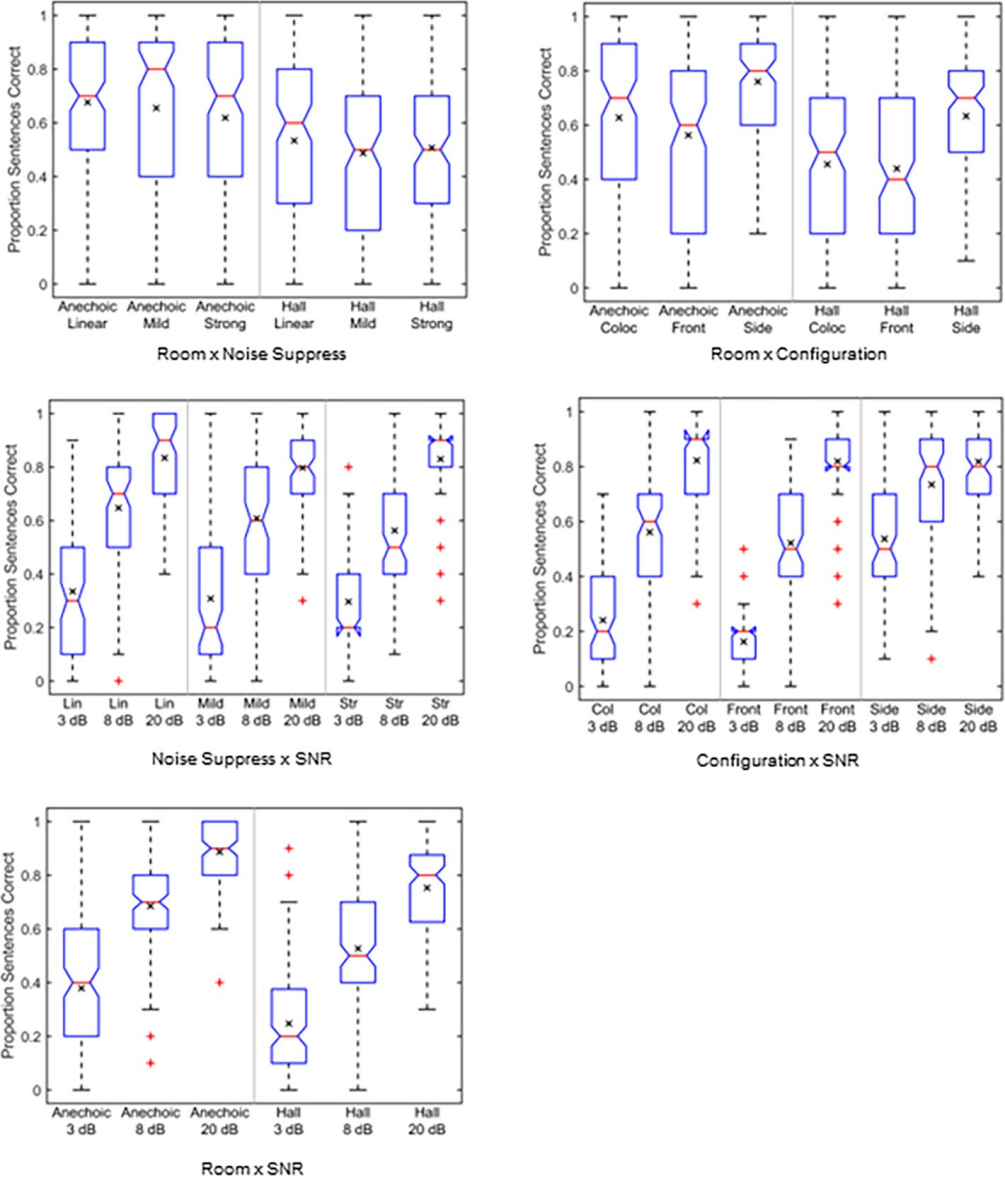
Box plots showing interactions for the NH listener group. The interactions are room × noise suppression, room × spatial configuration, suppression × SNR, room × SNR, and spatial configuration × SNR. The means are given by the × within each box. Data are averaged over repetitions for each subject.

The box plots in Fig 4 show distributions of intelligibility scores for interactions within the NH group. The interaction of room × noise suppression is shown in the top left panel. Intelligibility is lower for all three noise suppression settings in the hall than in the anechoic environment (*p* < 0.001, 0.84 ≤ *d* ≤ 1.07). For the anechoic presentation there are no significant differences between linear and mild (*p* > 0.999, *d* = 1.07) and between mild and strong (*p* = 0.394, *d* = 0.27), but intelligibility is significantly higher for linear than for strong (*p* < 0.01, *d* = 0.43). For the concert hall, there are no significant differences between any of the noise suppression conditions (*p* > 0.065, 0.15 ≤ *d* ≤ 0.35).

The interaction of room × spatial configuration is shown in the top right panel. Intelligibility for the side configuration is higher than for either the colocated or front in both rooms (*p* < 0.001, 0.99 ≤ *d* ≤ 1.47). Intelligibility is significantly higher for the colocated than for the front configuration for the anechoic room (*p* < 0.001, *d* = 0.49) but there is no significant difference for the hall (*p* > 0.999, *d* = 0.12). Intelligibility in the hall is lower than in the anechoic environment for all three spatial configurations (*p* < 0.001, 0.92 ≤ *d* ≤ 1.29).

Noise suppression × SNR is shown in the middle-left panel. Intelligibility at 20 dB SNR is greater than at 8 or 3 dB SNR and intelligibility at 8 dB SNR is greater than at 3 dB SNR for all three noise suppression settings (*p* < 0.001, 1.39 ≤ *d* ≤ 3.98). There are no significant differences between the linear, mild, and strong settings at any of the three SNRs (*p* > 0.999, 0.03 ≤ *d* ≤ 0.63). Room × SNR is shown in the lower left panel; intelligibility in the anechoic condition is significantly higher than for the hall at all three SNRs, and intelligibility at the higher SNRs becomes significantly higher for each step-wise increase in SNR (*p* < 0.001, 0.98 ≤ *d* ≤ 2.27) for both rooms.

Finally, spatial configuration × SNR is shown in the middle right panel. Again, intelligibility increases significantly for each upward step in SNR (*p* < 0.001, 0.62 ≤ *d* ≤ 2.68) for each configuration. At the 3 dB SNR, intelligibility is significantly higher for the side than for colocated or front configurations (*p* < 0.001, 2.22 ≤ *d* ≤ 2.80) and is higher for the colocated than for the front configuration (*p* = 0.004, *d* = 0.58). At the 8 dB SNR intelligibility is higher for the side than for colocated or front configurations (*p* < 0.001, 1.29 ≤ *d* ≤ 1.59) but not significantly different between the front and colocated presentation (*p* > 0.999, *d* = 0.30), while at 20 dB SNR there is no significant difference between any of the configurations (*p* > 0.999, 0.01 ≤ *d* ≤ 0.03).

### Pairwise comparisons: HI Group

**Fig 5:**
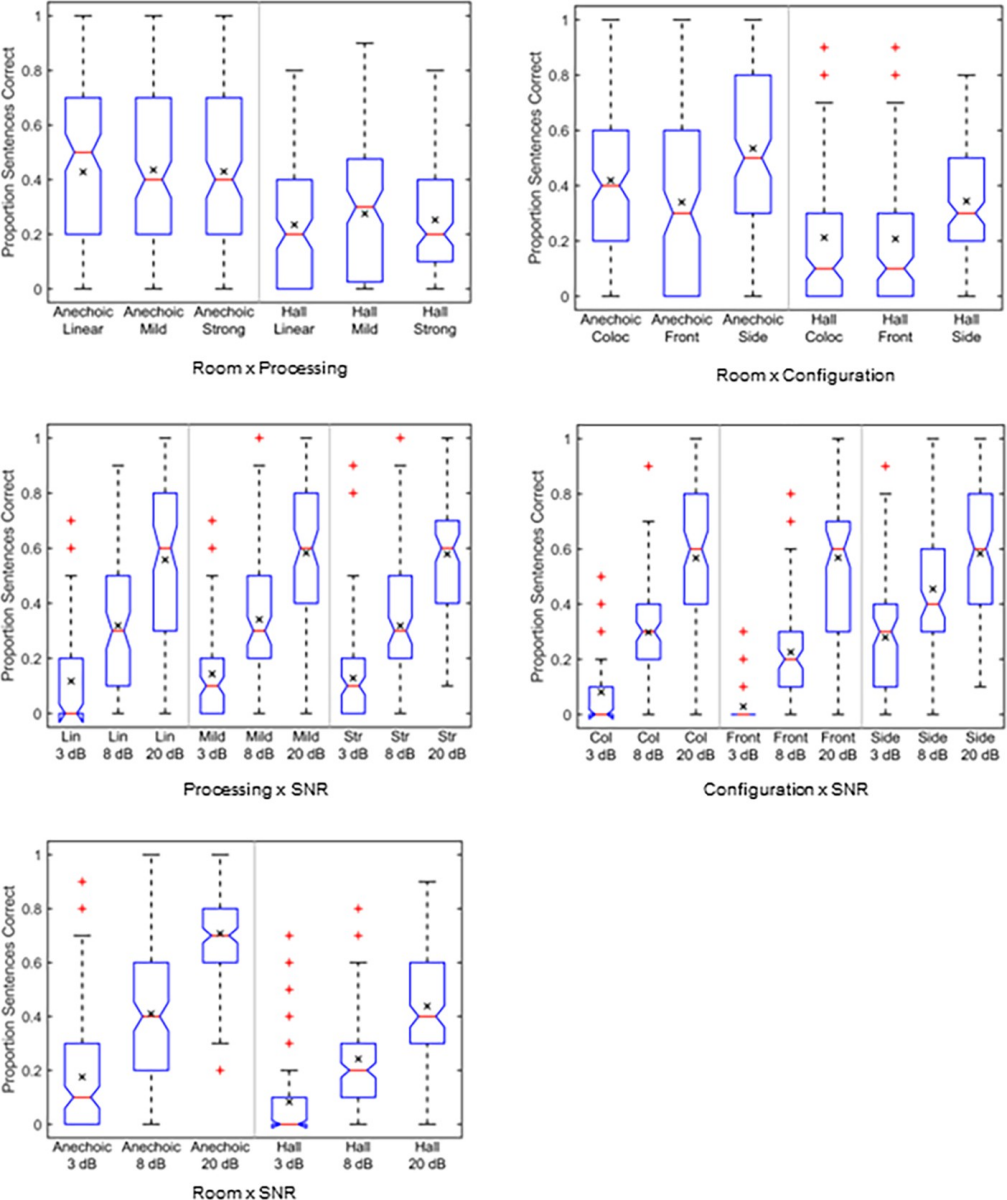
Box plots showing interactions for the HI listener group. The interactions are room × HA processing, room × spatial configuration, processing × SNR, room × SNR, and spatial configuration × SNR. The means are given by the × within each box. Data are averaged over repetitions for each subject.

The box plots in Fig 5 show distributions of intelligibility scores for interactions within the HI group. Room × HA processing is shown in the top left panel. Intelligibility is lower in the hall than in the anechoic environment for all three processing settings (*p* < 0.001, 1.11 ≤ *d* ≤ 1.34). However, there are no significant differences between the linear, mild, and strong settings in either room (*p* ≥ 0.310, 0.02 ≤ *d* ≤ 0.28).

Room × spatial configuration is shown in the top right panel. Intelligibility for the side configuration is higher than for either the colocated or front configurations in both rooms (*p* < 0.001, 0.80 ≤ *d* ≤ 1.35). Intelligibility for the colocated configuration is higher than for the front configuration for the anechoic presentation (*p* < 0.001, *d* = 0.55) but not for the hall (*p* > 0.999, *d* = 0.03). Intelligibility in the hall is lower than in the anechoic environment for all three spatial configurations (*p* < 0.001, 0.92 ≤ *d* ≤ 1.44).

HA processing × SNR is shown in the middle-left panel. Intelligibility at 20 dB SNR is greater than at 8 or 3 dB SNR and intelligibility at 8 dB SNR is greater than at 3 dB SNR for all three HA processing settings (*p* < 0.001, 1.32 ≤ *d* ≤ 3.12). There are no significant differences between the linear, mild, and strong settings at any of the three SNRs (*p* > 0.999, 0.01 ≤ *d* ≤ 0.17). Room × SNR is shown in the lower left panel; intelligibility in the anechoic condition is significantly higher than for the hall at all three SNRs, and intelligibility at the higher SNRs becomes significantly higher for each increase in SNR (*p* < 0.001, 0.64 ≤ *d* ≤ 2.07) for both rooms.

Finally, spatial configuration × SNR is shown in the middle right panel. As for the NH listener group, intelligibility increases significantly for each upward step in SNR (*p* < 0.001, 0.89 ≤ *d* ≤ 2.37) for each configuration. Intelligibility at the 3 dB SNR is significantly higher for the side configuration than for the colocated or front configurations (*p* < 0.001, 1.37 ≤ *d* ≤ 1.74) but is not significantly different between the colocated and front configurations (*p* = 0.482, *d* = 0.37). Intelligibility at the 8 dB SNR is significantly higher for the side configuration than for the colocated or front configurations (*p* < 0.001, 1.09 ≤ *d* ≤ 1.59) and higher for the colocated than for the front configuration (*p* = 0.030, *d* = 0.50). Intelligibility at the 20 dB SNR shows no significant differences between the three configurations (*p* > 0.999, 0.01 ≤ *d* ≤ 0.11).

## Discussion

### Spatial configuration

Surprisingly, the spatial configuration with a frontal target and a noise source on each side (front condition) was found as challenging, or even slightly more challenging, than the colocated condition where there is no spatial separation between the speech and noise sources. For example, significantly poorer intelligibility for the front as compared to the colocated condition was observed for the linear processing data plotted in Fig 3 and for the anechoic data plotted in the upper right-hand panels of Figs 4 and 5 for the NH and HI subjects, respectively. Having a noise masker with only limited envelope modulations on each side of the target speech would give minimal opportunity for better-ear glimpsing. However, the fact that the noise sources lead to ITDs different from the centered speech ITD should still allow for binaural unmasking that should provide an intelligibility advantage in the front condition compared to the colocated condition [3, 51, 52].

To further investigate this issue, the predicted spatial release from masking and the relative components of this release associated with better-ear glimpsing (BE) and binaural unmasking (BU) were computed using a binaural speech intelligibility model for non-stationary noise maskers and NH listeners [53]; this model is available open-access as vicente2020nh within the Auditory Modelling Toolbox [54]. Note that this model is not able to predict the potential deleterious effect of reverberation temporally smearing the target speech (which can be evaluated here by comparing the data in the anechoic and reverberant colocated conditions).

Without requiring any fitting to the data, the model can predict the spatial release from masking due to BE-only, BU-only or to the combination of these two binaural effects, using as inputs the speech and noise signals at each ear. These signals were simulated for a NH listener, considering the frequency responses of the HA and vent, but assuming no further processing in the HA (i.e. linear condition). The spatial release from masking was evaluated in six conditions (2 rooms × 3 spatial configurations) using the anechoic colocated condition as a reference. It is expressed in dB and corresponds to the predicted difference in speech reception threshold (SRT, the SNR for 50% intelligibility) between the tested and reference conditions.

**Fig 6:**
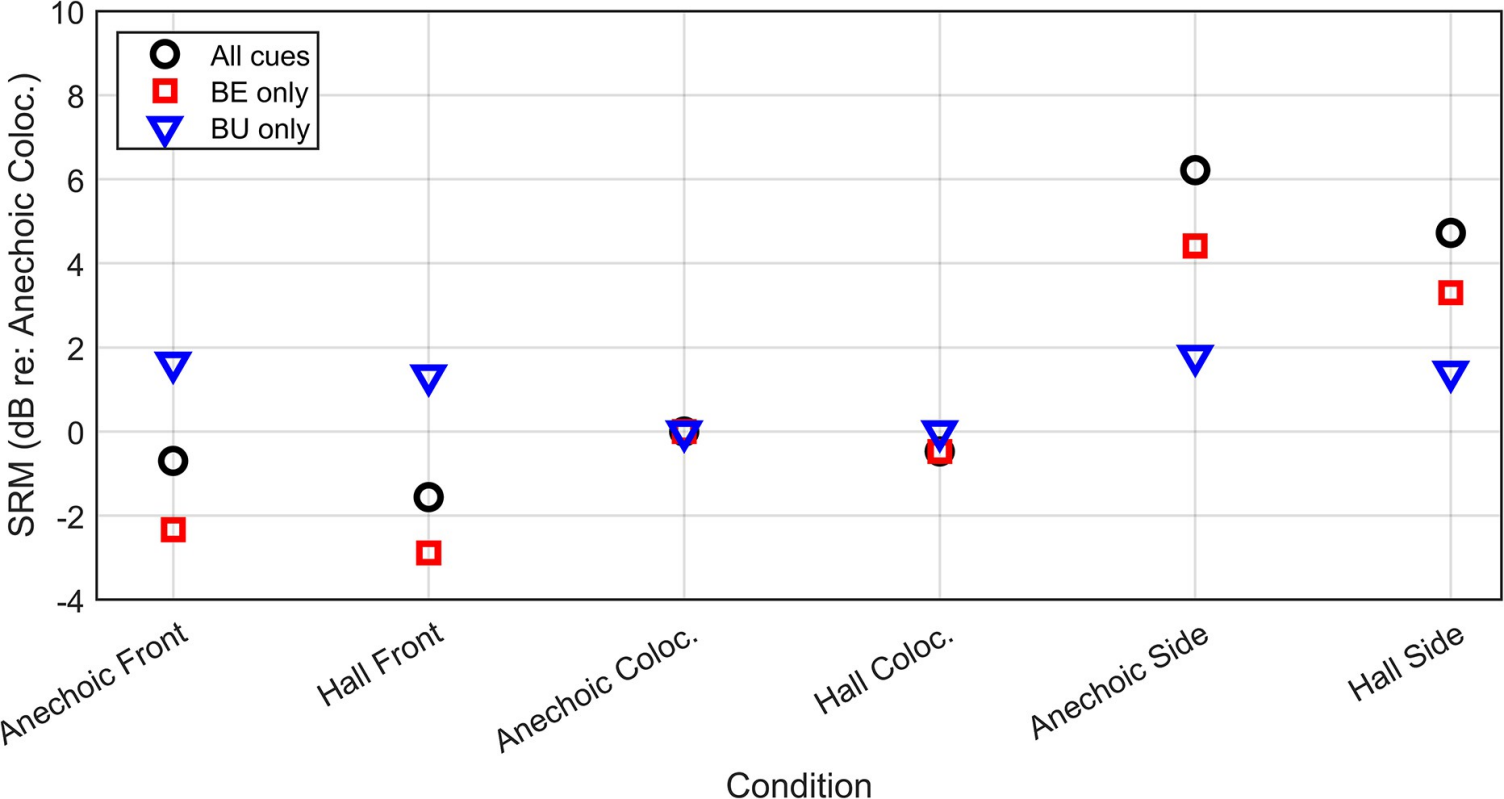
Predicted spatial release from masking for an NH listener. The predictions are for the 3 spatial configurations and 2 rooms tested in the study assuming BE-only, BU-only, or both binaural mechanisms (“All cues”) available. The anechoic colocated condition was used for the 0-dB reference. The predictions were computed using the vicente2020mh model proposed by Vicente and Lavandier (2020).

The input signals used to compute the predictions were prepared according to the model instructions [54]. The predictions presented in Fig 6 were computed using 50 realizations of the masker signals in each of the six tested conditions. The target was identical in all conditions and represented by averaging 50 target sentences. The averaging was accomplished by discarding the first 45 ms of each sentence and then truncating each sentence to the duration of the shortest target sentence, thereby ensuring that only overlapping continuous portions of speech were used for the predictions. These target and masker inputs were calibrated to the level of the corresponding target and masker signals during the experiment (at 0 dB SNR). The model was applied on these input signals and predictions were averaged across the 50 masker realizations.

The predictions presented in Fig 6 are consistent with what is observed in the data: the spatial release from masking, by definition 0 dB for the reference condition, is positive (providing an intelligibility benefit) in the side/asymmetric masker condition, thanks to both BE and BU that provide for about 4 dB and 2 dB of release, respectively. These releases are reduced in reverberation, as expected from the literature [55]. However, the spatial release from masking is slightly negative in the front/symmetric masker condition, as also observed in the data. Rather than being due to an absence of BE and BU, the model predicts that there is still a BU advantage of about 2 dB in this condition, but that the predicted BE release is negative (below -2 dB). Thus the model predicts that the noises cause more masking when they are placed symmetrically apart from the frontal speech target compared to when they are colocated with this target.

**Fig 7:**
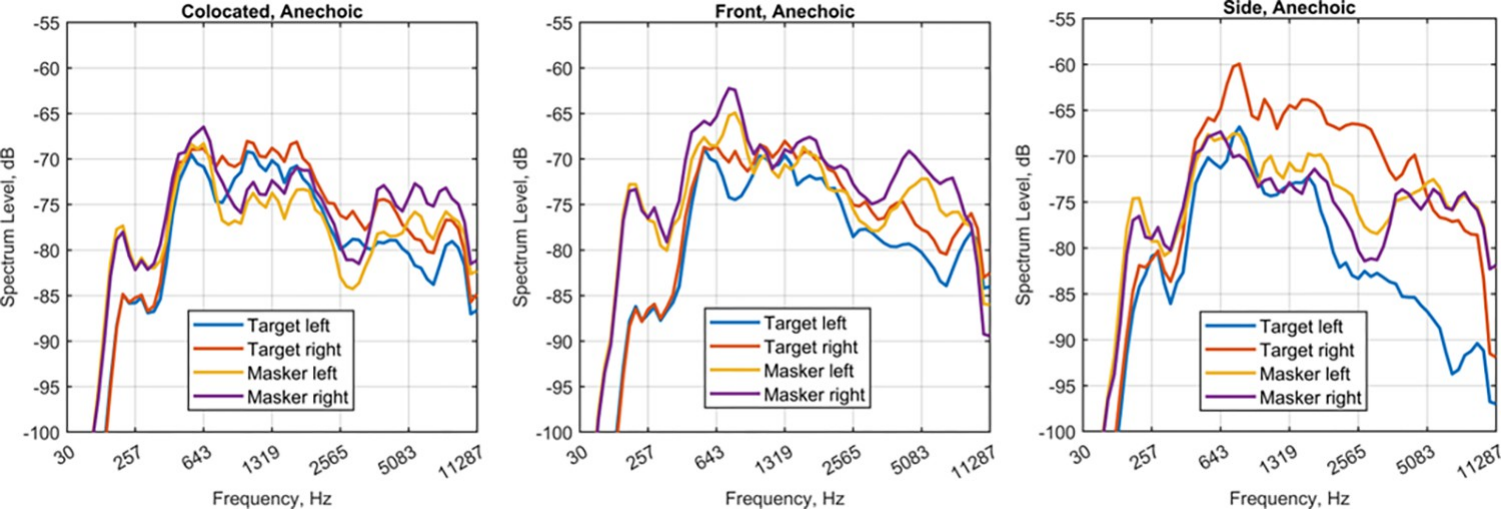
Mean speech and noise gammatone spectra at each ear in the three tested spatial configurations (colocated, front, side). The speech spectrum is obtained by computing the spectrum at the output of a gammatone filter bank (identical to the one used in the binaural model vicente2020nh) for the target input signal used in the BU/BE model predictions (average of 50 sentences). The noise spectrum is obtained by averaging the corresponding spectra of the 50 masker input signals.

The long-term spectra of the signals in the anechoic conditions, shown in Fig 7, confirm an increase in masker levels below about 1 kHz at both ears when the maskers are in the front/symmetric condition compared to the colocated condition. Thus the absence of spatial release in the front/symmetric condition is most probably associated with the particular HRTFs used in the present study. Asymmetries have been documented in the KEMAR pinnae [56] and torso [23] magnitude frequency responses, and the positioning of the amplifiers, cables, and measurement equipment in the test chamber was not symmetrical. The spectra of the frontal sources in the anechoic colocated condition highlight the asymmetries in the HRTFs simulated with the test recording set-up, especially in that the right-ear levels are several dB above the left-ear levels.

### Noise and reverberation

**Fig 8:**
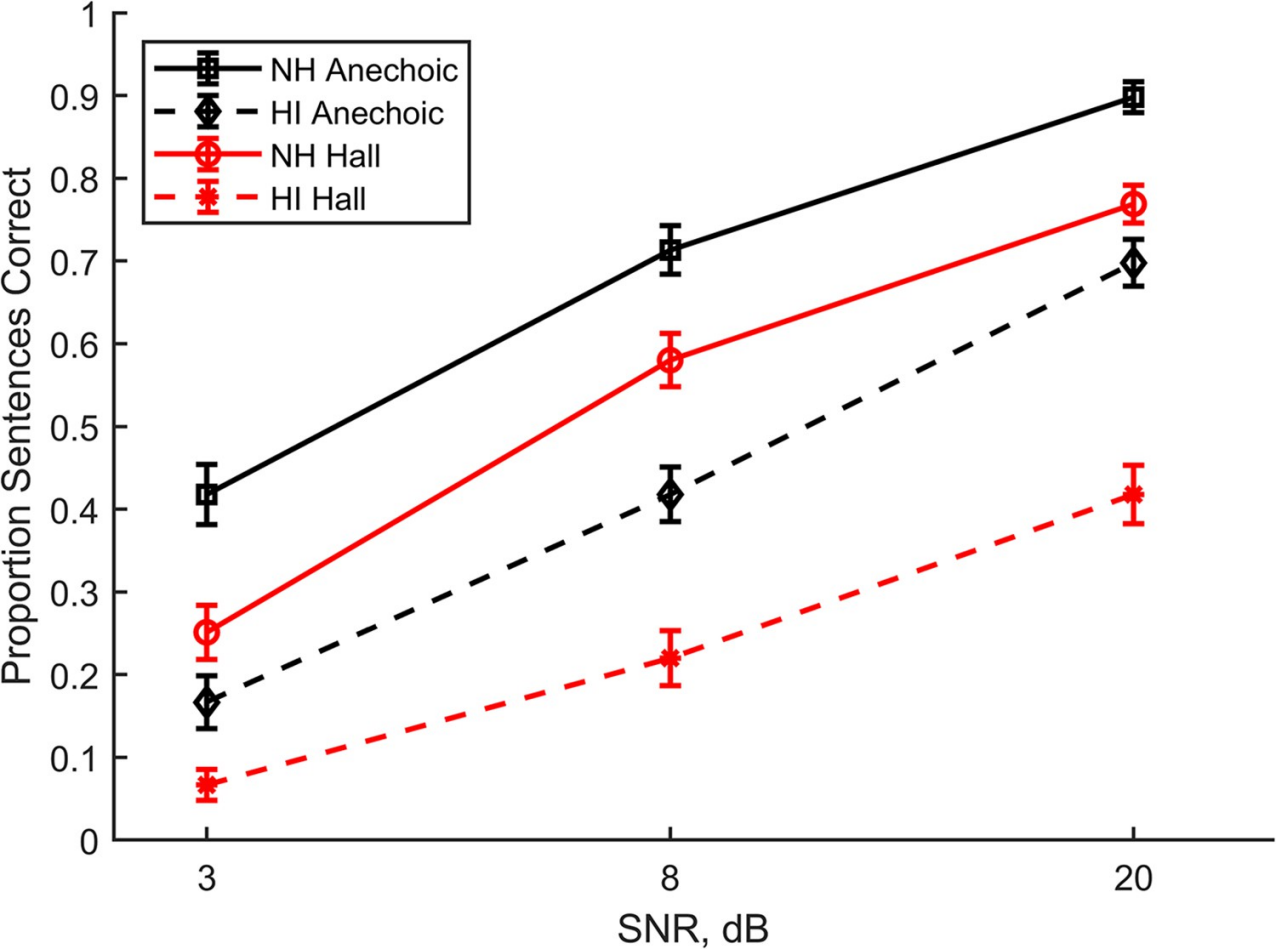
**Intelligibility as a function of room and SNR.** The curves are for the NH and HI listeners in the linear processing condition. The error bars represent the standard error of the mean.

Several previous studies have explored intelligibility in additive noise, reverberation, and the combination of noise and reverberation for NH and HI listeners [8, 12–18]. The linear processing condition in the present study allowed the comparison of NH and HI results for noise and reverberation when linear amplification was used to compensate for the hearing loss. These results were extracted from Figs 4 and 5 and replotted in Fig 8. They are consistent with the results reported in the literature; intelligibility decreases with decreasing SNR and decreases in reverberation for both NH and HI listeners. Direct comparisons between intelligibility scores from different studies are difficult, however, given the differences in test stimuli and scoring; for example, scoring IEEE sentences in terms of proportion keywords correct will yield different indicated intelligibility than scoring the same materials in terms of proportion complete sentences correct [57].

### Noise suppression and HA processing

The interaction of reverberation, WDRC release time, and working memory for HI listeners was investigated [13] in which IEEE sentences, scored as the number of keywords correct, were presented in quiet using a simulated room and simulated hearing aid. That study found that intelligibility decreased with increased RT and decreased with shorter WDRC release times. The closest corresponding conditions in the present paper were the anechoic room and concert hall for the mild HI processing. The data in Fig 5 show a significant reduction in intelligibility for the HI listeners for the hall compared to the anechoic room for the mild processing condition, which agrees with the earlier result.

A simulation analysis of several noise suppression algorithms [27] predicted that there would be no intelligibility improvement for NH or HI listeners when the estimated noise level used in the processing was the average over the entire utterance rather than the instantaneous intensity in each time-frequency cell. The noise suppression used in the present paper was the same as the spectral subtraction used in the simulation analysis when the estimated noise level was computed as the average over the stimulus duration. The noise suppression × SNR results for the NH listeners shown in Fig 4 show no significant intelligibility improvement when the noise suppression is used. The NH suppression × room data in the same figure show a significant reduction in intelligibility for the strong condition (12 dB maximum) compared to the linear processing. These listener results agree with the simulation analysis [27] that indicates minimal expected benefit for many simple noise suppression algorithms in hearing aids.

This conclusion is reinforced by HI subject results [58], which showed that there was no significant effect of noise suppression for anechoic speech intelligibility but a significant detriment in intelligibility for speech in reverberation when using spectral subtraction having a slowly-varying estimate of the noise level [59]. It is possible, however, that using noise estimation having a faster reaction to the variations in noise intensity over time than used in this paper could yield some improvement in intelligibility. A study [18] using a faster noise estimator [60] found that intelligibility for both NH and HI listeners was improved for the noise suppression combined with syllabic WDRC but was reduced when combined with slow WDRC for both NH and HI listeners.

The mild and strong HA processing conditions differ primarily in the inclusion of frequency compression in the strong condition for the HI listeners. However, the HI results in Fig 5 show no significant differences between the HA processing conditions at any of the three SNRs and no significant differences between processing in either of the two rooms. In particular, the lack of any significant intelligibility difference between the mild and strong HA processing implies that there is little intelligibility improvement at the sentence level due to the frequency compression algorithm. This finding is consistent with previous studies that have used the same frequency compression algorithm but with monaural stimulus presentation. One such study [30] found that at a cutoff frequency of 2 kHz, frequency compression had minimal effect on keyword intelligibility in IEEE sentences independent of the frequency compression ratio for both NH and HI listener groups. At lower cutoff frequencies, intelligibility for both groups decreased with increasing frequency compression ratio. A second study [61] found that frequency compression having a cutoff frequency of 1.5 kHz had only a small effect on keyword intelligibility in quiet and in noise for HI listeners, but reducing the cutoff frequency to 1 kHz greatly reduced intelligibility. The cutoff frequencies and frequency compression ratios used in the present paper are, in general, less pronounced than those used in the two studies cited above, so smaller changes in intelligibility would be expected.

### Limitations

The ability to generalize the results of this paper may be limited by some aspects of the stimulus generation and HA processing. The room simulation used KEMAR HRIR recordings, which would be expected to produce reduced intelligibility compared to using individual HRIRs [62, 63]. The KEMAR recordings also preclude head motion while listening to speech in noise and reverberation, which could also reduce intelligibility compared to free head motion [64]. The room simulation also used loudspeakers arrayed in the azimuthal plane, which eliminated any potential interaction of intelligibility with floor or ceiling reflections and the associated elevation cues that would occur in an actual room.

An additional signal processing consideration is that the HA processing conditions combined multiple algorithms into the mild and strong conditions. This grouping provided realistic HA settings but makes it difficult to factor out the contributions of the individual algorithms considered in some of the previous studies. Since the focus of the experiment reported in this paper was speech intelligibility, the differences in speech quality that can be caused by noise, reverberation, and nonlinear signal processing were not evaluated. However, previous work in our laboratory [26] has shown that binaural quality and intelligibility have a positive association with each other; both decrease with reductions in signal fidelity caused by additive noise and with the nonlinear distortion associated with hearing-aid processing. It was also observed that as signal fidelity decreased, quality ratings changed at a slower rate than intelligibility scores.

## Conclusions

This paper presented speech intelligibility results for realistic listening conditions combined with realistic HA signal processing. Rather than present a detailed analysis of the effects of varying just one or two parameters, this paper focused on the interactions of many parameters–hearing loss group, room acoustics, speech and noise source configuration, amount of additive noise, noise suppression, WDRC, and frequency compression.

Conclusions from this study include:

- An unexpected result was observed concerning the effects of the spatial configuration: The colocated configuration tended to have higher intelligibility than the front configuration. This result, however, was explained by a model of binaural unmasking and better ear glimpsing calculated for the anechoic signals recorded at KEMAR’s ears.
- The reductions in intelligibility for additive noise and reverberation were shown to be significant and consistent with results reported in the literature.
- The noise suppression results reported in this paper are complementary with those reported in previous studies. The noise suppression here showed no significant improvement in speech intelligibility for noise suppression based on the noise level averaged over the duration of the speech utterance. However, previous studies used a slowly-varying or a rapidly-varying noise estimator, with the rapidly-varying noise estimate suggesting a possible benefit in noise. The results taken across these studies suggest that, as predicted in the simulation study of [27], noise suppression benefit depends strongly on the noise estimation procedure.
- HA processing results for the HI listeners showed no significant difference between linear, mild, and strong processing in either room or at any of the three SNRs despite the increasing amount of signal modification. There was no apparent overall benefit for the syllabic WDRC implemented in this study and no overall benefit for frequency compression combined with WDRC. This lack of observed benefit illustrates the difficulty in designing more effective hearing aids and determining the effects of HA algorithms when several processing algorithms are operating at the same time and interacting with each other and the acoustic environment.
- The dataset acquired in this study covered a wide range of conditions for NH and HI listeners. It will be a valuable resource for developing future binaural models of speech intelligibility that include spatial configuration, noise, room, hearing loss, and signal processing effects. The stimulus sound files and the subject responses have been uploaded to the Open Science Framework (OSF) public-domain repository. The NH data are available at https://osf.io/nf23j and the HI data are available at https://osf.io/yz64u.
